# Endothelial cells activate the cancer stem cell‐associated *NANOGP8* pathway in colorectal cancer cells in a paracrine fashion

**DOI:** 10.1002/1878-0261.12071

**Published:** 2017-06-06

**Authors:** Rui Wang, Rajat Bhattacharya, Xiangcang Ye, Fan Fan, Delphine R. Boulbes, Ling Xia, Lee M. Ellis

**Affiliations:** ^1^ Department of Surgical Oncology The University of Texas M.D. Anderson Cancer Center Houston TX USA; ^2^ Department of Gastroenterology Research The University of Texas M.D. Anderson Cancer Center Houston TX USA; ^3^ Department of Molecular and Cellular Oncology The University of Texas M.D. Anderson Cancer Center Houston TX USA

**Keywords:** cancer stem cells, colorectal cancer, endothelial cell, microenvironment, NANOG, paracrine

## Abstract

In colorectal cancer (CRC), cancer stem cells (CSCs) have been hypothesized to mediate cell survival and chemoresistance. Previous studies from our laboratory described a role for liver parenchymal endothelial cells (LPECs) in mediating the CSC phenotype in CRC cells in a paracrine/angiocrine fashion. The objectives of this study were to determine whether endothelial cells (ECs) from different organs can induce the CSC phenotype in CRC cells and to elucidate the signaling pathways involved. We treated a newly developed CRC cell line (HCP‐1) and established CRC cell lines (HT29 and SW480) with conditioned medium (CM) from primary ECs isolated from nonmalignant liver, lung, colon mucosa, and kidney. Our results showed that CM from ECs from all organs increased the number of CSCs, as determined by sphere formation, and protein levels of NANOG and OCT4 in CRC cells. With the focus of further elucidating the role of the liver vascular network in mediating the CSC phenotype, we demonstrated that CM from LPECs increased resistance to 5‐fluorouracil in CRC cells. Moreover, we showed that LPEC CM specifically induced *NANOGP8* expression in CRC cells by specific enzyme digestion and a luciferase reporter assay using a vector containing the *NANOGP8* promoter. Lastly, we found that LPEC CM‐induced *NANOGP8* expression and sphere formation were mediated by AKT activation. Our studies demonstrated a paracrine role for ECs in regulating the CSC phenotype and chemoresistance in CRC cells by AKT‐mediated induction of *NANOGP8*. These studies suggest a more specific approach to target CSCs by blocking the expression of *NANOGP8* in cancer cells.

**Abbreviations**
5‐FU: 5‐fluorouracil
CM: conditioned medium
CRC: colorectal cancer
CSC: cancer stem cells
EC: endothelial cells
HCP‐1: human CRC primary cells
LPEC: liver parenchymal endothelial cell

## 1 Introduction

There are now 10 drugs approved by the Food and Drug Administration for treating patients with metastatic colorectal cancer (mCRC). However, the disease remains the second‐leading cause of cancer‐related death in the United States (American Cancer Society, accessed December 2016). With median survival being only ~ 2.5 years, patients with mCRC will often develop drug resistance to systemic therapy within one year of treatment (Fakih, 2015; Sanz‐Garcia *et al*., 2016). Having a better understanding of the regulation of chemoresistance in CRC cells would lead to new opportunities for therapeutic interventions and improve outcomes of patients with mCRC.

In mCRC and many other cancers, cancer stem cells (CSCs) have been suggested to mediate cell survival, metastasis, and resistance to chemotherapy (Botchkina, 2013; Flemming, 2015; Todaro *et al*., 2010). These functional properties associated with CSC cells, sphere‐forming ability, and chemoresistance are often referred as ‘the CSC phenotype’. CSCs, make up about 5% of cancer cells, are a distinct population of cancer cells that share many features with non‐neoplastic stem cells. Stem cell‐associated genes are highly expressed in CSCs, and activation of stem cell‐associated pathways (such as Wnt/β‐catenin, NOTCH, NANOG, and SHH/GLI) promotes the CSC phenotype in cancer cells (Kreso and Dick, 2014). However, the mechanisms of regulating stem cell‐associated gene expression and the CSC phenotype in CRC cells have not yet been fully elucidated. Our laboratory described a role for endothelial cells (ECs) in mediating the CSC phenotype in CRC cells in a paracrine/angiocrine fashion. We showed that liver parenchymal endothelial cells (LPECs) secrete soluble Jagged‐1 in conditioned medium (CM) that, in turn, activates NOTCH signaling and increases the CSC phenotype of CRC cells. With increased number of CSCs in the population, CRC cells demonstrated increased chemoresistance and became more metastatic to the liver (Lu *et al*., 2013).

In agreement with our findings, other groups who conducted preclinical studies have described angiocrine signaling, ECs secreting soluble factors, and cytokines in a paracrine manner. Results from their studies demonstrated that ECs regulate hematopoietic stem cell development (Butler *et al*., 2010), liver cancer cells’ invasion (Wang *et al*., 2013), and survival and growth in a variety of cancer cell types (Butler *et al*., 2010). Moreover, several studies showed that CSCs in glioblastoma are enriched at the tumor perivascular niche (Gilbertson and Rich, 2007), and the tumor microenvironment (including ECs) is essential to maintain CSCs in glioblastoma (Lathia *et al*., 2015). Similar CSC‐promoting features of ECs have also been described in other cancer types including head and neck cancer (Krishnamurthy *et al*., 2010) and lung cancer (Liborio *et al*., 2015). Studies also showed that the angiocrine profiles of distinct organ‐specific ECs are widely divergent and that a specific angiocrine profile is required to mediate desired effects on the local cell population, including stem cells (Rafii *et al*., 2016). However, the scope of most studies was limited to the effect of few EC cell lines on a limited number of target cells (e.g., human umbilical vein cells’ effect on neural stem cells, testicular ECs’ effect on spermatogonial stem cells, and liver ECs’ effect on hepatocytes). No extensive studies have compared the effects of ECs from different organs on the CSC phenotype.

The aim of this study was to determine whether ECs from different organs can induce the CSC phenotype in CRC cells. We showed that CM from ECs from all organs studied contained secreted factors that increased the number of colorectal CSCs, as determined by the sphere formation assay, and increased chemoresistance in CRC cells. Moreover, we showed that CM from ECs from various organs activated the CSC‐promoting NANOG pathway in CRC cells. These findings demonstrate the role of ECs in establishing a prosurvival microenvironment for CRC cells in the colon and in other organs of metastasis.

## 2 Materials and methods

### 2.1 Cell culture

The CRC cell lines SW480 and HT29 were purchased from American Type Culture Collection (ATCC, Manassas, VA, USA). The human CRC primary cell line (HCP‐1) and endothelial cell (EC) lines were established in our laboratory (Lu *et al*., 2013). CRC cells were cultured in MEM supplemented with 5% FBS (Atlanta Biologicals, Atlanta, GA, USA), vitamins (1 ×), nonessential amino acids (1 ×), penicillin/streptomycin antibiotics (1 ×), sodium pyruvate (1 ×), and l‐glutamine (1 ×), all from Invitrogen (Carlsbad, CA, USA). ECs were cultured in EC Growth Medium MV2 (PromoCell, Heidelberg, Germany) supplemented with 10% human serum (Atlanta Biologicals). All cell lines were used within 10 passages. All cell lines were authenticated in every six months by short tandem repeat test from the Characterized Cell Line Core Facility at M.D. Anderson Cancer Center. For HCP‐1 cells and ECs established in our laboratory, genomic DNA from the original tissue samples were used for authentication.

### 2.2 Reagents

The PI3K inhibitor wortmannin was from Sigma (St. Louis, MO, USA). 5‐Fluorouracil (5‐FU) was obtained from the pharmacy at The University of Texas MD Anderson Cancer Center. *NANOG/NANOGP8*‐specific siRNAs (si‐2: 5′‐CAGCUGUGUGUACUCAAUG, si‐3: 5′‐UGGAACAGUCCCUUCUAUA) and a validated control siRNA were obtained from Sigma and were transiently transfected by using Lipofectamine 2000 (Invitrogen) according to the manufacturer's instructions.

### 2.3 Conditioned medium

Equal numbers of CRC cells or ECs were cultured in growth medium with 1% FBS (0.1 × 10^6^ cells·mL^−1^) for 48 h. CM was harvested and centrifuged at 4000 ***g*** for 5 min to remove cell debris. CM from each CRC cell line was used as controls.

### 2.4 Western blotting

Cell lysates were processed and run in SDS/PAGE gels as described previously (Wang *et al*., 2014, 2016). Antibodies used to detect α‐tubulin, NANOG/NANOGP8 (sc‐134218), and HRP‐conjugated β‐actin were from Santa Cruz Biotechnology (Santa Cruz, CA). All other antibodies were from Cell Signaling (Beverly, MA, USA). Sizes of bands were estimated based on protein standards (Bio‐Rad, Hercules, CA, USA) on the membrane.

### 2.5 Sphere formation assay

CRC cells were pretreated with wortmannin, or transfected by siRNAs in selected experiment. CRC cells were incubated with CM for 48 h, and then, single suspended cells were plated 100 per well in ultra‐low‐attachment 96‐well plates (BD Biosciences, San Jose, CA, USA) in 1 : 1 CM and standard sphere‐forming medium [serum free DMEM/F‐12 supplemented with 1xB27 serum substitute, 20 ng·mL^−1^ human recombinant EGF, and 20 ng·mL^−1^ basic FGF, all from Invitrogen (Reynolds *et al*., 1992)]. Wortmannin was added to the medium during sphere formation. For siRNA, cells were transfected with siRNA once prior sphere formation. After 7–14 days, spheres that were larger than 50 μm in diameter in each well were counted as described previously (Wang *et al*., 2016).

### 2.6 CRC cell spheres for cell death and viability

CRC cells were plated 1000 per well in ultra‐low‐attachment six‐well plates in sphere formation medium. After 7–10 days when the average size of spheres reached 50 μm in diameter, all spheres were evenly distributed to ultra‐low‐attachment plates and then treated without or with 5‐FU in 1 : 1 standard sphere formation medium and CM. For western blotting, spheres were treated with 5‐FU and CM in ultra‐low‐attachment six‐well plates for 48 h and collected for protein lysate. For cell viability, spheres were treated with 5‐FU and CM in ultra‐low‐attachment 96‐well plates for 72 h. Then, MTT substrate (0.25% in PBS; Sigma) was added to spheres for 1 h at 37 °C. Spheres were then collected, washed with PBS, and dissolved in 100 μL DMSO. Optical density was measured at 570 nm, and relative MTT was presented as % of control.

### 2.7 Cell apoptosis assays

CRC cells were treated with or without 5‐FU in CM for 48 h. Protein levels of apoptotic markers were assessed by western blotting. Cell apoptosis was determined using the FITC Annexin V Apoptosis Detection Kit I from BD Biosciences. Single suspended cells were double‐stained with FITC Annexin V and propidium iodide and analyzed by fluorescence‐activated cell sorting (FACS). Double‐positive cells were counted as apoptotic cells and presented as a percentage of the total cells.

### 2.8 Luciferase reporter assay

The assay was performed as described previously (Wang *et al*., 2012, 2014) with the DUO‐GLO Luciferase Assay System (Promega, Madison, WI, USA). Luciferase reporter constructs and the *Renilla* loading control construct were cotransfected by Lipofectamine 2000. After transfection, cells were recovered in normal growth medium overnight and then incubated in CM for 24 h before measurement. *OCT4*‐Luc was a gift from Shinya Yamanaka (Addgene plasmid #17221) (Takahashi *et al*., 2007), and *NANOG*‐Luc was a gift from Ren‐he Xu (Addgene plasmid #25900) (Xu *et al*., 2008). *NANOGP8*‐Luc was constructed by cloning the upstream 5‐kb sequence of human *NANOGP8* into pMCS‐Red Luc vector (ThermoFisher, Rockford, IL, USA) with primers (forward: 5′‐TTAACGGGGTACCGAGACAACACAAGGAACTAGTGATGCAGGTCATAAACGC, reverse: 5′‐GGCACGGGGATCCCGTTAAAATCCTGGCAAGATGTGCTTTGTTAAACAG) and restriction enzymes *Kpn*I and *Bam*HI (New England BioLabs, Ipswich, MA, USA), respectively.

### 2.9 Reverse transcriptase polymerase chain reaction (RT‐PCR)

RT‐PCR was performed as described previously (Wang *et al*., 2014). cDNA transcription and PCR were performed using SuperScript III First‐Strand RT‐PCR Kit (Invitrogen). Primers designed by Primer Blast (NCBI Primer BLAST) were used to detect following human genes: *OCT4* (forward: 5′‐GGCCACACGTAGGTTCTTGA, reverse: 5′‐CTCCCCACTAGGTTCAGGGA) and *HPRT1* (forward: 5′‐GCGTCGTGATTAGCGATGATGAAC, reverse: 5′‐CCTCCCATCTCCTTCATGACATCT). Primers for human *NANOG/NANOGP8* were designed to produce a ~ 300‐bp cDNA fragment flanking the nucleotide 144 from the starting codon (forward: 5′‐CCGACTGTAAAGAATCTTCACC, reverse: 5′‐GACAGAAATACCTCAGCCTCC). Sizes of bands were estimated based on expected product length from primer design and DNA ladders (Sigma) on the gel.

### 2.10 *Alw*NI digestion

Restriction enzyme *Alw*NI was from New England BioLabs. cDNAs of *NANOG* and *NANOGP8* containing nucleotide 144 were amplified by RT‐PCR with primers described above. cDNA fragments were purified by a PCR Purification Kit (QIAGEN, Valencia, CA, USA) and subjected to *Alw*NI digestion according to the manufacturer's instructions.

### 2.11 Statistical analysis

All quantitative data were reproduced in at least three independent experiments with multiple measures in each replicate. The resulting data were expressed as means ± standard error of the mean (SEM) and were considered to be significantly different when *P* < 0.05 by two‐tailed Student's *t*‐test.

## 3 Results

### 3.1 CM of ECs from distinct organs increased sphere formation in CRC cells

**Figure 1:**
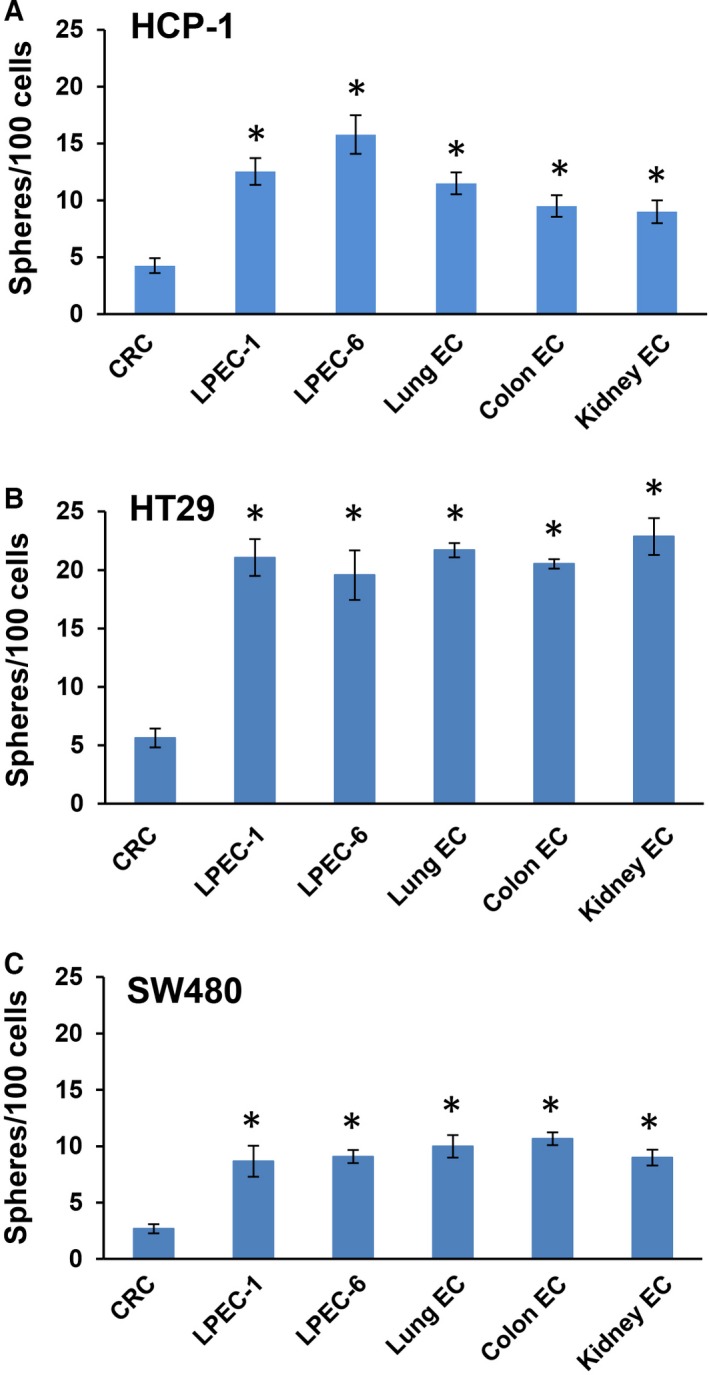
CM of ECs from distinct organs increased sphere formation in CRC cells. (A–C) CRC cells were treated either with their own control CM (CRC) or with CM of ECs from liver (LPEC‐1, LPEC‐6), lung, colon mucosa (colon), or kidney. The sphere formation assay showed increased spheres formed per 100 cells per well. Mean ± SEM, *P < 0.05, *t*‐test.

To determine whether ECs from different organs, including liver, promote the CSC phenotype of CRC cells, our laboratory has established an additional liver EC line (LPEC‐6) and ECs from other healthy organs and tissues (lung, colon mucosa, and kidney). LPEC‐1 cells were also used as an internal control to validate our previous findings. The CSC phenotype of CRC cells was firstly assessed by sphere formation assays, a validated assay for determining the number of CSCs in the cancer cell population (Fan *et al*., 2014; Lee *et al*., 2016). CM containing secreted factors was harvested from each cell line and used for sphere formation assays. Compared with control CM from CRC cells themselves, CM from LPECs and from ECs from other organs individually increased sphere formation in the HCP‐1 cell line (two‐ to threefold) and in the HT29 and SW480 cell lines (both ~fourfold) (Fig. 1).

### 3.2 CM of ECs from distinct organs activated NANOG signaling pathway in CRC cells

Our laboratory reported that the NOTCH pathway was involved in LPEC‐1 CM induction of the CSC phenotype in CRC cells (Lu *et al*., 2013). To determine whether other CSC‐associated pathways are also activated by EC CM, we performed an unbiased qPCR array comparing the gene expression profiles of CRC cells that were incubated either in their own CM or in LPEC‐1 CM. In agreement with our previous findings, *HES‐1* expression (downstream target of the NOTCH pathway) was increased by LPEC‐1 CM. Moreover, we found that another CSC‐associated NANOG pathway was also activated [increased *NANOG* and *OCT4* (also known as *POU5F1*) expression] in LPEC‐1 CM‐treated CRC cells, whereas other CSC‐associated genes [such as *GLI*, CTNNB1 (β‐catenin) *TCF4*,* LGR5,* and *BMI*] remained unchanged (data not shown).

NANOG was first recognized as a key regulator for keeping the pluripotency of embryonic stem cells by forming a transcription complex with its downstream target OCT4 (Amoils, 2006). It has then been characterized to promote the CSC phenotype in different cancer types, including CRC (Jeter *et al*., 2015; Sun *et al*., 2014). In humans, NANOG proteins can be encoded by either *NANOG* or *NANOGP8*, a retrogene derived from *NANOG*, located on different chromosomes (Fairbanks *et al*., 2012). Studies showed that normal colon mucosa has low or undetectable *NANOG* and *NANOGP8*, whereas colorectal tumor tissues and cancer cells have elevated *NANOGP8* expression with no changes in *NANOG* (Ishiguro *et al*., 2012; Jeter *et al*., 2011; Zhang *et al*., 2013). These studies also suggested that *NANOG8* is responsible for regulating the CSC phenotype in CRC and other cancer cells.

**Figure 2:**
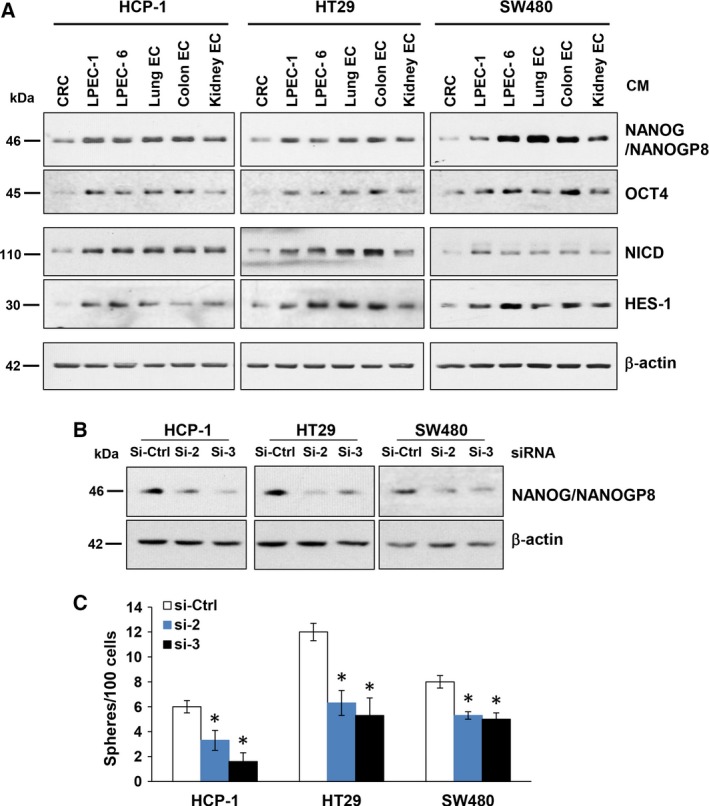
CM of ECs from distinct organs activated the NANOG pathway in CRC cells. (A) CRC cells were treated either with their own control CM (CRC) or with CM from ECs from distinct organs. Western blotting shows increased protein levels of NANOG/NANOGP8, OCT4, cleaved NOTCH1 (NICD), and HES‐1. β‐Actin was used as the loading control. (B,C) CRC cells were transiently transfected with *NANOG/NANOGP8*‐specific siRNAs (si‐2, si‐3). (B) Western blotting shows decreased NANOG/NANOGP8 protein levels. β‐Actin was used as the loading control. (C) Decreased sphere formation per 100 cells/well. Mean ± SEM, *P < 0.05, *t*‐test. Antibody and siRNAs could not distinguish proteins or mRNA expressed by *NANOG* and *NANOGP8* in CRC cells.

The qPCR array we performed could not determine whether *NANOG* or *NANOGP8* was induced by LPEC‐1 CM. We first validated the activation of NOTCH and NANOG pathways by western blotting (Fig. 2A). In CRC cells, the protein levels of cleaved NOTCH1 (NICD) and HES‐1 (NOTCH pathway), NANOG/NANOGP8, and its downstream target OCT4 (NANOG pathway) were dramatically increased by CM from LPECs and ECs from different organs. The protein bands were labeled as NANOG/NANOGP8 because the antibodies used could not determine whether the detected proteins were encoded by *NANOG* or *NANOGP8* mRNA. We also confirmed that proteins involved in other CSC‐associated pathways (such as GLI and β‐catenin) were not altered by CM of ECs (data not shown).

To confirm the importance of the NANOG pathway in promoting the CSC phenotype in CRC cells, we used two different siRNAs targeting the common sequences of *NANOG* and *NANOGP8* for gene knockdowns *in vitro*. After confirming siRNA knockdown of NANOG/NANOGP8 by western blotting (Fig. 2B), transfected CRC cells were subjected to sphere formation assays. Results in Fig. 2C showed that both siRNAs significantly decreased the number of CSCs, as determined by the sphere formation assay, in CRC cell lines. Our data suggest that ECs from different organs were all capable of increasing the number of colorectal CSCs and activating the NANOG pathway in a paracrine fashion. Because liver is the most common site of metastases in patients with mCRC, we focused on examining the effects of LPECs on CRC cells in the following studies.

### 3.3 CM from LPECs increased chemoresistance of CRC cells

**Figure 3:**
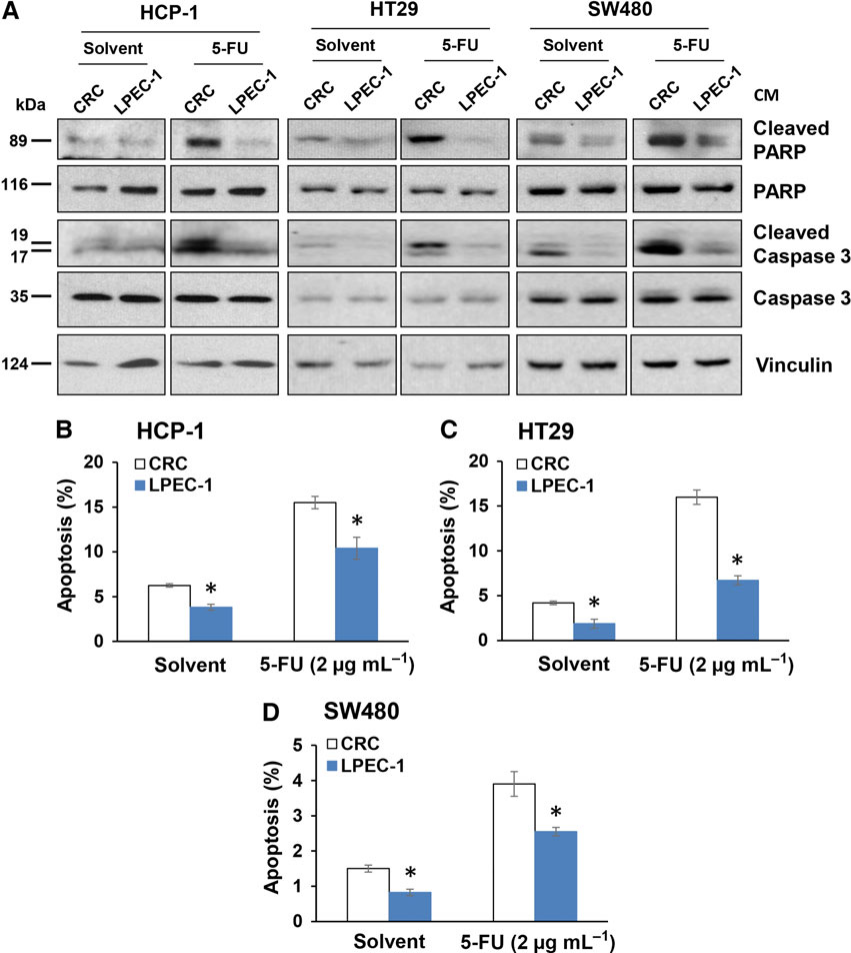
LPEC‐1 CM increased chemoresistance in CRC cells. CRC cells were treated without (solvent) or with fluorouracil (5‐FU) in either their own CM (CRC) or liver EC CM (LPEC‐1). (A) Western blotting showed that LPEC‐1 CM decreased 5‐FU‐induced protein levels of apoptotic markers (cleaved PARP and cleaved caspase 3) in CRC cells. Vinculin was used as the loading control. (B–D) Apoptotic cells in CRC cells were determined by FACS with Annexin V and propidium iodide double staining and were presented as a percentage of the total cells. Mean ± SEM, *P < 0.05, *t*‐test.

To determine whether the observed induction of the CSC phenotype by CM from ECs also affected the response of CRC cells to chemotherapy, we treated CRC cells with a clinically relevant dose (2 μg·mL^−1^) of 5‐FU (Gamelin *et al*., 2008) in either control CM or CM from LPEC‐1. Apoptosis of CRC cells was first examined by western blotting for protein levels of apoptotic markers (Fig. 3A). When cells were incubated in their own control CM, 5‐FU treatment dramatically increased the protein levels of cleaved PARP and cleaved caspase 3 in CRC cells. In contrast, CRC cells cultured with LPEC‐1 CM had lower levels of cleaved PARP and cleaved caspase 3 compared with levels in control groups, and the addition of 5‐FU did not increase the protein levels of these apoptotic markers. We then determined the number of apoptotic cells in the population by FACS analysis with Annexin V and propidium iodide double staining (Fig. 3B–D). 5‐FU significantly increased the percentage of apoptotic cells in control CM, but the induction of apoptosis by 5‐FU was blocked when CRC cells were incubated in LPEC‐1 CM. Similar results were observed when the same experiment was performed with LPEC‐6 CM (Fig. S1). Moreover, the effect of LPEC‐1 CM on chemoresistance of CRC cells was also assessed in spheres formed by HCP‐1 cells. After HCP‐1 cells formed spheres, subsequent incubation with LPEC‐1 CM not only decreased 5‐FU‐induced apoptosis, as determined by western blotting, but also increased the protein levels of NANOG/NANOGP8 in HCP‐1 spheres (Fig. S2A). In addition, the MTT assay showed that while 5‐FU significantly decreased cell viability in HCP‐1 spheres in control HCP‐1 CM, incubation with LPEC‐1 CM made CRC cells more resistant to 5‐FU as demonstrated by significantly higher cell viability (Fig. S2B). These data suggest that LPEC‐1 CM blocked 5‐FU‐induced apoptosis of CRC cells not only in 2D‐cultured cells, but also in cancer cell spheres.

### 3.4 CM from LPECs induced *NANOGP8* expression in CRC cells

**Figure 4:**
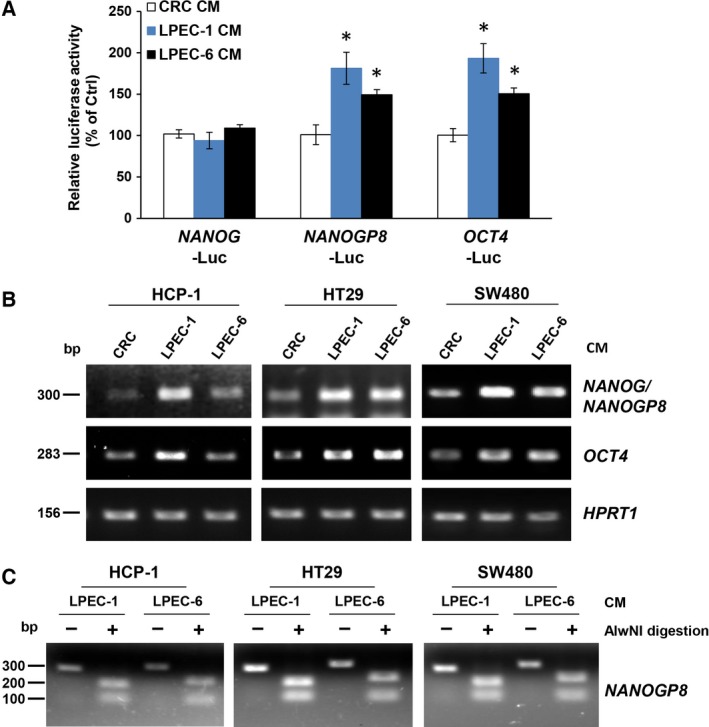
LPEC CM increased *NANOGP8* expression in CRC cells. CRC cells were treated with their own control CM (CRC) or liver EC CM (LPEC‐1 or LPEC‐6). (A) Luciferase reporter assay showed increased promoter activity of *NANOGP8* and *OCT4* genes, but not *NANOG*. Mean ± SEM, *P < 0.05, *t*‐test. (B) RT‐PCR showed increased mRNA levels of *NANOG/NANOGP8* and *OCT4* genes. *HPRT1* was used as the loading control. Primers recognized and amplified both human *NANOG* and *NANOGP8* mRNA. (C) *NANOG/NANOGP8* cDNA fragments were amplified by RT‐PCR and digested without (−) or with (+) *Alw*NI restriction enzyme. All cDNA fragments were susceptible to *Alw*NI digestion.

We performed luciferase reporter assays to further validate the EC CM induction of NANOG/NANOGP8 and OCT4 in CRC cells. We obtained luciferase reporter constructs containing the promoter regions of human *NANOG* and *OCT4* genes (Takahashi *et al*., 2007; Xu *et al*., 2008) and constructed the reporter construct containing the 5‐kb genomic sequence upstream of human *NANOGP8*. Due to difficulty in transient transfection, only SW480 cells were used in the experiment (Fig. 4A). In agreement with the data shown in Fig. 2, transcription of the *OCT4* gene in CRC cells was significantly increased by CM from LPEC‐1 (twofold) and LPEC‐6 (~ 60%). However, the transcription of *NANOG* was not changed by LPEC CM treatment; instead, that of *NANOGP8* was significantly increased in CRC cells by CM from LPEC‐1 (twofold) and LPEC‐6 (60%). These results showed for the first time that CM of LPECs specifically induced *NANOGP8*, but not *NANOG*, and its downstream target *OCT4* in CRC cells. After the luciferase reporter assay, we then performed semiquantitative RT‐PCR to confirm that incubation of CM from both LPECs increased the mRNA levels of *OCT4* and *NANOG/NANOGP8* in all CRC cell lines tested (Fig. 4B).

To determine the specific expression of *NANOGP8* in CRC cells, we digested the RT‐PCR‐amplified cDNA fragments with *Alw*NI, a method described previously to distinguish mRNA transcripts of *NANOG* and *NANOGP8* (Fig. S3) (Ishiguro *et al*., 2012; Zhang *et al*., 2013). We found that all CRC cell lines used in our studies expressed high levels of *NANOGP8* with undetectable *NANOG* (data not shown). More importantly, we showed that the RT‐PCR products from LPEC CM‐treated CRC cells were all digested by *Alw*NI (Fig. 4C), suggesting that the increased mRNA levels we detected in Fig. 4B were transcribed by *NANOGP8*. Together, these data suggest that all CRC cell lines used in these studies expressed *NANOGP8* with undetectable *NANOG* and that the treatment of LPEC CM had specifically increased *NANOGP8* expression in CRC cells.

### 3.5 AKT mediated LPEC‐1 CM Induction of *NANOGP8* in CRC cells

**Figure 5:**
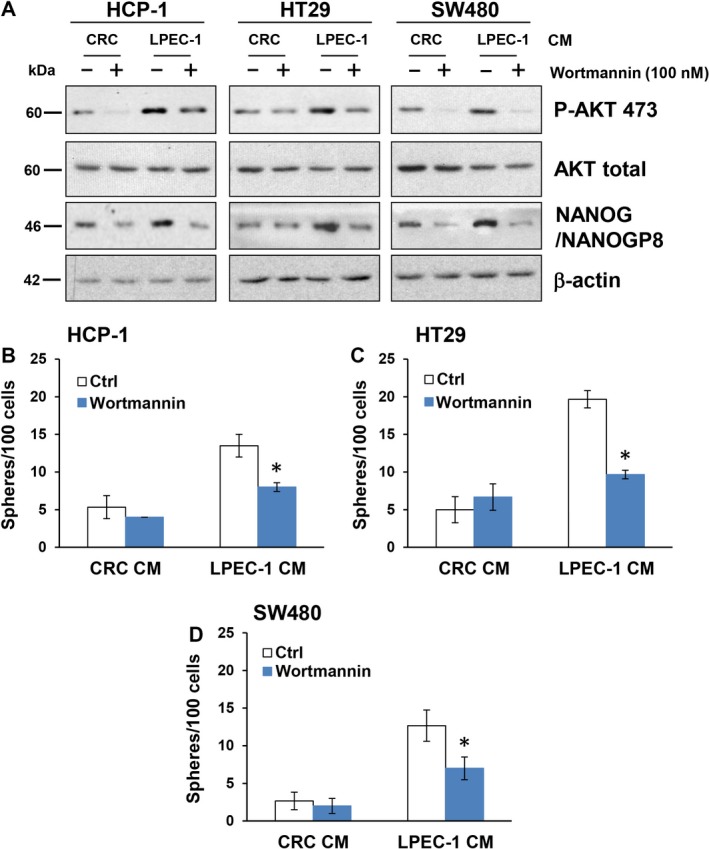
AKT mediated LPEC‐1 CM activation of *NANOGP8* and the CSC phenotype in CRC cells. CRC cells were treated without (− or Ctrl) or with (+ or wortmannin) the PI3K inhibitor wortmannin and in either control CM (CRC) or liver EC CM (LPEC‐1). (A) CRC cells were treated with wortmannin overnight and then with CM for 30 min. Western blotting shows that LPEC‐1 CM‐induced AKT phosphorylation (P‐AKT 473) and NANOG protein levels were decreased by the inhibitor. β‐Actin was used as the loading control. (B–D) LPEC‐1 CM‐induced sphere formation in CRC cells was decreased by wortmannin. Mean ± SEM, *P < 0.05, *t*‐test.

To elucidate the mechanism of *NANOGP8* induction by CM from LPECs, we examined several mechanisms that had been reported for regulating NANOG expression in different cell types. We detected no activation of these pathways [TGF‐β/SMAD (Xu *et al*., 2008), IL‐6/STAT3 (Kim *et al*., 2013), and OCT4 stabilization by AKT (Lin *et al*., 2012)] in CRC cells treated by CM from LPEC (data not shown). However, we noticed that AKT was activated by CM from EC lines from liver and other organs (Fig. S4). Further analysis revealed that inhibition of AKT phosphorylation by the PI3K inhibitor wortmannin decreased the NANOG protein levels in CRC cells. Moreover, CM from LPEC‐1 did not increase levels of NANOG proteins (encoded by *NANOGP8*) in the presence of wortmannin (Fig. 5A). The effect of AKT inhibition on the CSC phenotype was then determined by sphere formation (Fig. 5B–D). Although LPEC‐1 CM significantly induced sphere formation in all three CRC cell lines, adding the PI3K inhibitor to LPEC‐1 CM partially blocked the induction of sphere formation in CRC cells.

## 4 Discussion

The importance of the tumor microenvironment has been discussed in depth in many different types of cancers (Quail and Joyce, 2013). This study focused on elucidating the role of ECs, a critical component of the microenvironment, in CRC cell survival and growth. We sought to determine the paracrine role of ECs in regulating the CSC phenotypes of CRC cells and the specific pathways involved. We used LPECs to represent the liver EC microenvironment, and this is clinically relevant because the most common site of CRC distant metastasis is the liver. ECs from lung, colon mucosa, and kidney were also used to compare the effects of ECs from different organs on CRC cells. Our data showed for the first time that CM from ECs from several different organs increased the number of CSCs, as determined by sphere formation, and activated the CSC‐associated NANOG pathway in CRC cells.

Focusing on studying the liver vascular microenvironment, we used LPECs to demonstrate that CM from these ECs increased chemoresistance in CRC cells, as LPEC CM‐treated CRC cells had decreased 5‐FU‐induced apoptosis compared to control cells. Moreover, using luciferase reporter assays and *Alw*NI digestion, we showed that LPEC CM specifically induced the *NANOGP8* expression in CRC cells. We do not know whether CM from ECs from lung, colon mucosa, and kidney increased NANOG protein levels by inducing the transcription of *NANOG* or *NANOGP8*. However, because only *NANOGP8* was highly expressed in CRC cells and tumor tissues (Ishiguro *et al*., 2012; Jeter *et al*., 2011), we believe that the induced NANOG proteins in CRC cells by CM from ECs from different organs were encoded by *NANOGP8*. Of note, the *OCT4*‐luc construct contains the promoter regions of the *OCT4* gene on chromosome 6, which is different from that of *OCT4* pseudogenes (Suo *et al*., 2005). Therefore, liver EC CM induced the transcription of *OCT4* gene, not its pseudogenes.

We showed that ECs from different organs all had ability to increase the number of CSCs in CRC cells and increased proteins levels of NANOG and OCT4. However, CRC metastases are most commonly found in liver and lungs, but rarely found in other visceral organs (such as kidney). This discrepancy can be explained that the blood circulation brings most blood from small and large intestines directly to the liver, then to the lungs. As a result, circulating cancer cells that were detached from primary CRC tumors will likely adhere and grow in the liver and lungs before they reach other organs. Also, it is possible that, as described by the seed and soil hypothesis (Langley and Fidler, 2011), stromal cells (other than ECs) in different organs also play important roles in metastasis. Metastatic cancer cells are more likely to survive and grow in the microenvironments of liver and lungs. Due to the logistics of the ability to harvest significant amounts of nonmalignant tissues for ECs harvest, we used kidney as an extra source of tissue for isolating ECs as residual tissue after surgical resection was large enough. Thus, the use of ECs from the kidney was done for proof‐of‐principle and was not intended to be of clinical relevance. This study, as well as prior studies from our laboratory (Lu *et al*., 2013), focused on ECs from liver, the most common site of distant metastases in patients with CRC.

To elucidate the mechanism of regulating *NANOGP8* expression in CRC cells, we have examined several mechanisms (TGFβ/SMAD, IL‐6/STAT3, and OCT4 protein degradation) that were reported to regulate the expression of NANOG proteins in different cell types. The original studies characterizing these pathways did not determine whether the described mechanisms were regulating *NANOG* or *NANOGP8* expression. The fact that we detected no change in these pathways when CRC cells were treated with CM from LPECs (data not shown) suggested that these pathways are not involved in LPEC CM induction of *NANOGP8* in CRC cells. Furthermore, we examined β‐catenin/TCF4 and c‐Myc pathways that were predicted to regulate *NANOGP8* transcription by an earlier study analyzing the *NANOGP8* promoter region (Jeter *et al*., 2015). Our unpublished data showed that the protein levels of c‐Myc and β‐catenin (by western blotting) and *TCF4* transcription activity (by luciferase reporter assay) were not altered by LPEC CM treatment. These findings suggest that LPEC CM induced *NANOGP8* expression in CRC cells through a mechanism that has not been characterized before.

In contrast to the above studies, we found that AKT was activated by LPEC CM and mediated the increased *NANOGP8* expression and number of CSCs in CRC cells, as determined by sphere formation. We sought to elucidate the mechanism of AKT activation in CRC cells by examining many pathways that have been reported for regulating AKT in cancer cells [such as EGFR, IGFR, FGFR, and MET (Mayer and Arteaga, 2016)], and found that none of these pathways were activated in CRC cells by CM from LPECs (data not shown). The mechanisms of LPEC CM activation of AKT and induction of *NANOGP8* in CRC cells remain to be elucidated. Further comprehensive studies are required to understand EC angiocrine signaling for increasing the CSC phenotype, and to elucidate the mechanism of AKT regulation of *NANOGP8* expression in CRC cells.

## 5 Conclusion

This study demonstrated a paracrine role of ECs in increasing the number of CSCs, as determined by sphere formation, and subsequent chemoresistance in CRC cells via activating the NANOG pathway. Inhibiting the NANOG pathway may potentially decrease the CSC phenotype in CRC cells and sensitize cancer cells to chemotherapy. Several clinical trials have been initiated to study NANOG inhibitors (such as BBI608 and its derivatives) in combination with chemotherapy for treating mCRC and other advanced malignancies. The development of NANOG inhibitors has been limited due to severe toxicities caused by off‐target effects. Our studies suggested a potential alternative strategy of inhibiting the NANOG pathway by blocking the gene expression of a more specific component of the NANOG pathway, *NANOGP8*, in cancer cells.

## Author Contributions

RW involved in conception and design, collection and assembly of data, data analysis and interpretation, and manuscript writing. RB and XY involved in data analysis and interpretation. FF collected and assembled the data. DRB involved in data analysis and interpretation, and manuscript editing. LX provided general technical support. LME provided financial support, and involved in conception and design, data analysis and interpretation, and final approval of the manuscript.

## Supporting information


**Fig. S1.** LPEC‐6 CM increased chemoresistance in CRC cells.

**Fig. S2.** LPEC‐1 CM increased NANOG protein levels and chemoresistance in HCP‐1 cell spheres.

**Fig. S3.** Schematic model of distinguishing *NANOG* and *NANOGP8*.

**Fig. S4.** CM of ECs from distinct organs activated AKT in CRC cells.
