# Supplementary figures and images for: Endothelial cells activate the cancer stem cell‐associated NANOGP8 pathway in colorectal cancer cells in a paracrine fashion
Source: Mol Oncol. 2017 Jun 6;11(8):1023–34. doi: 10.1002/1878-0261.12071 (PMC5537915; doi:10.1002/1878-0261.12071)

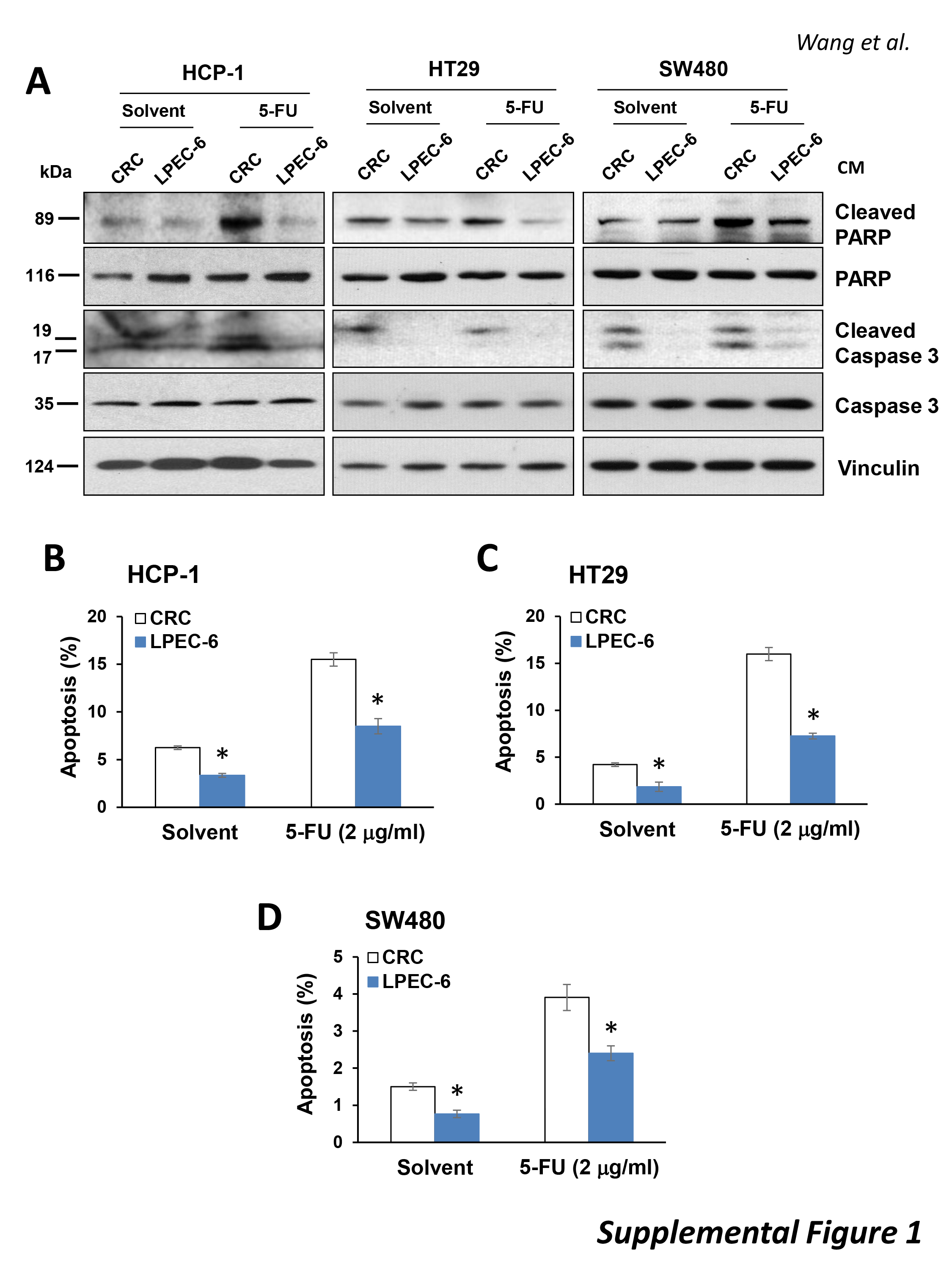

Supplement: Supplementary file 1 — Fig. S1. LPEC‐6 CM increased chemoresistance in CRC cells. [file MOL2-11-1023-s001.jpg]

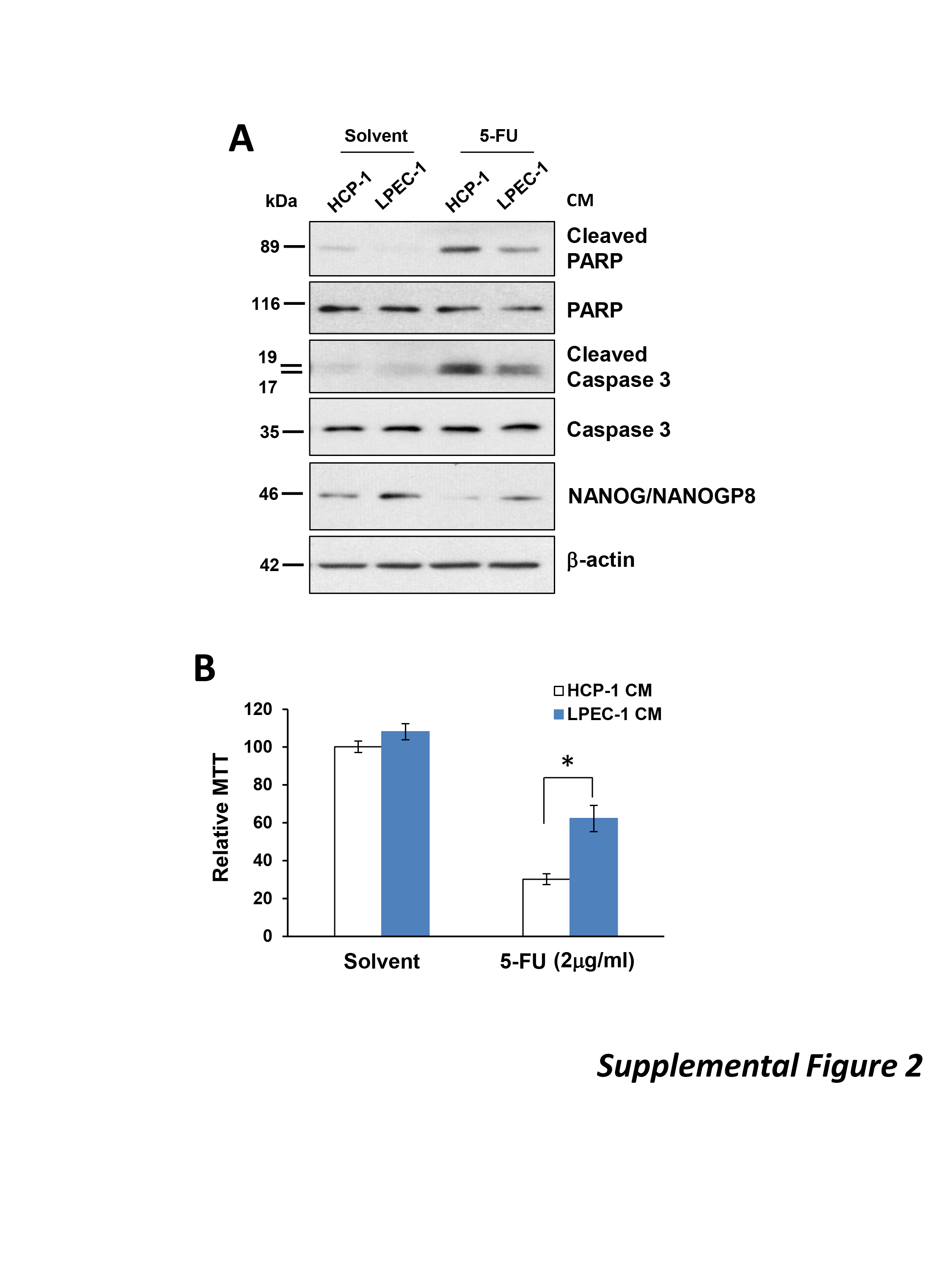

Supplement: Supplementary file 2 — Fig. S2. LPEC‐1 CM increased NANOG protein levels and chemoresistance in HCP‐1 cell spheres. [file MOL2-11-1023-s002.jpg]

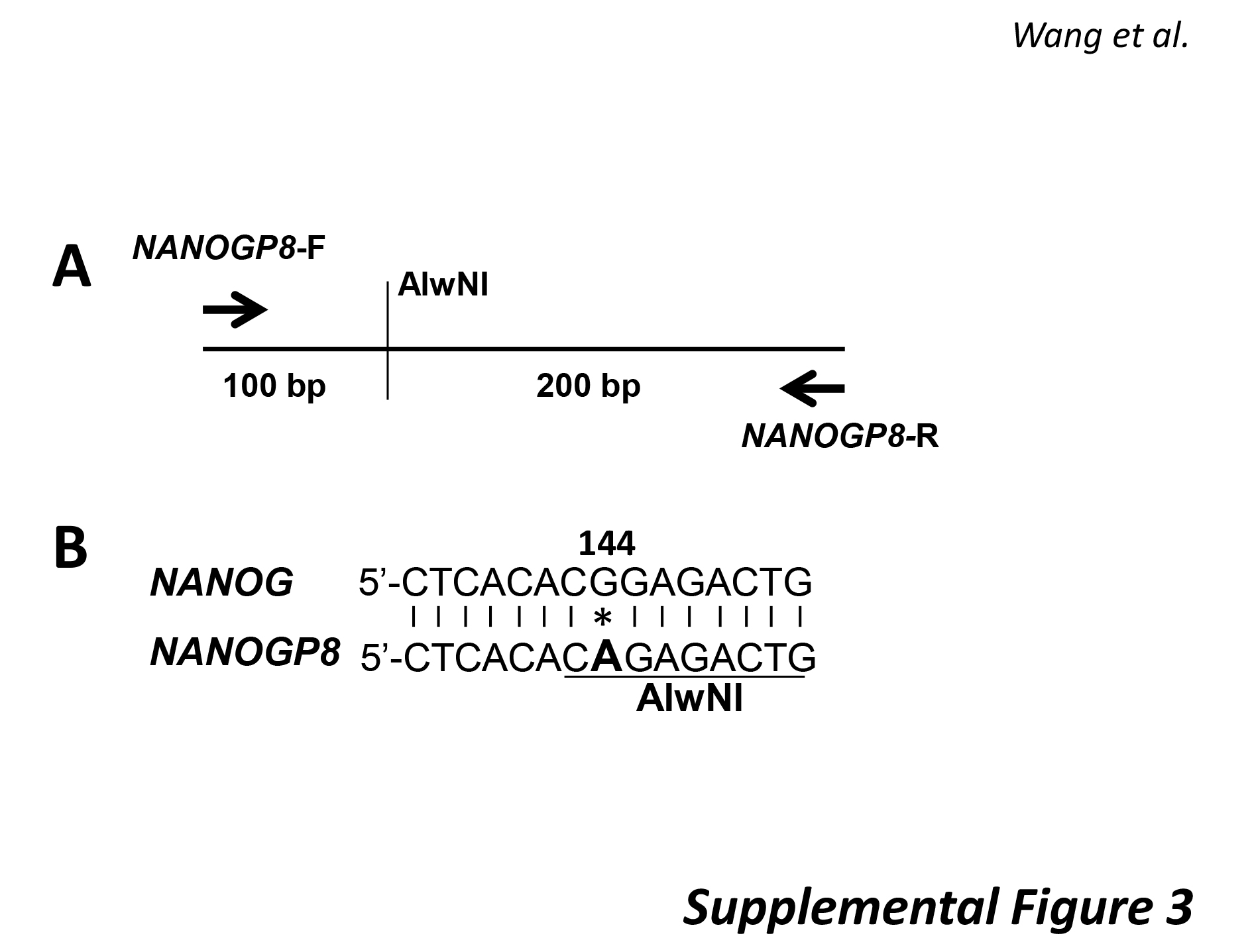

Supplement: Supplementary file 3 — Fig. S3. Schematic model of distinguishing NANOG and NANOGP8. [file MOL2-11-1023-s003.jpg]

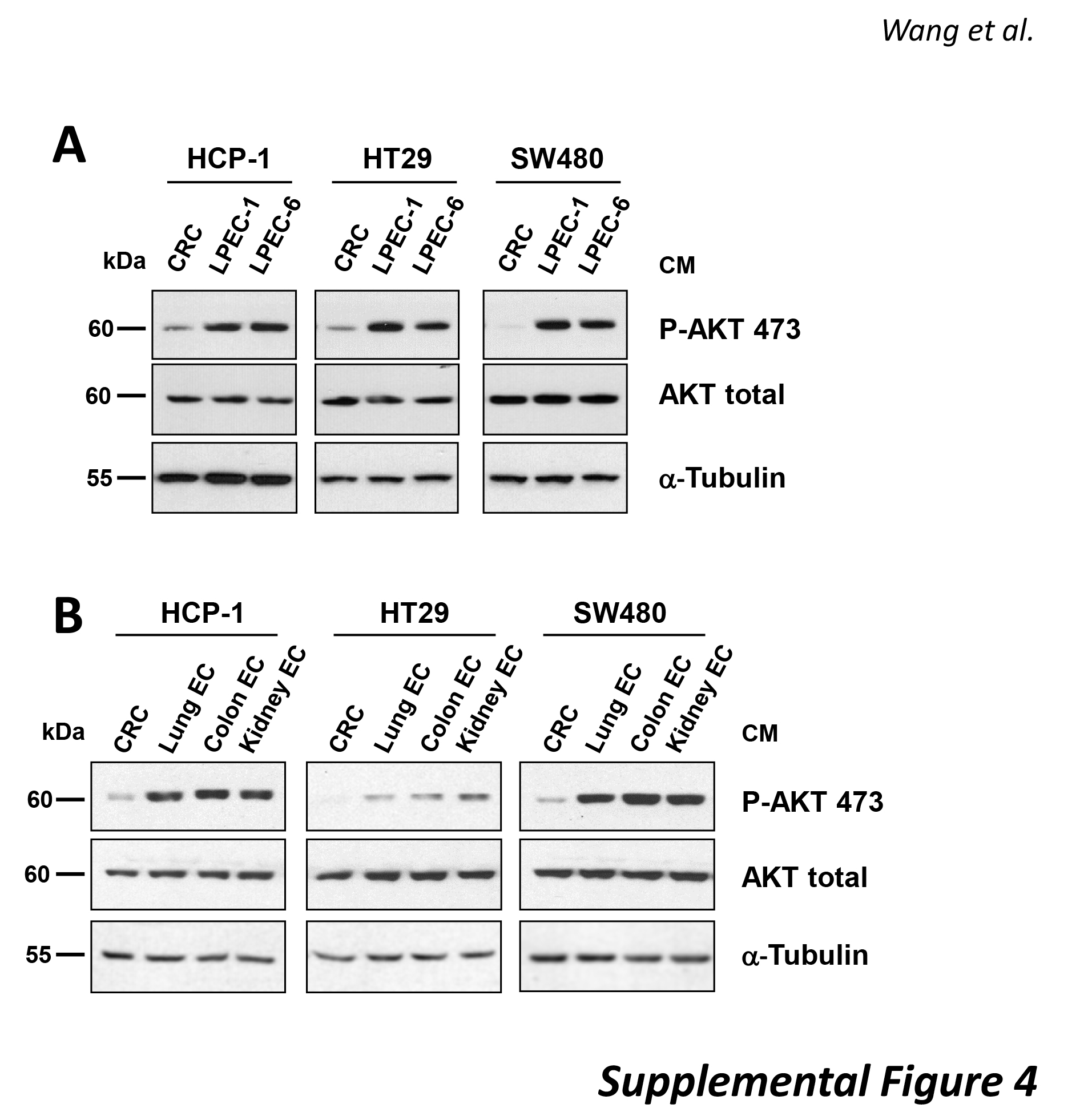

Supplement: Supplementary file 4 — Fig. S4. CM of ECs from distinct organs activated AKT in CRC cells. [file MOL2-11-1023-s004.jpg]
